# Eco-Physiological Responses of *Avicennia marina* (Forssk.) Vierh. to Trace Metals Pollution via Intensifying Antioxidant and Secondary Metabolite Contents

**DOI:** 10.3390/metabo13070808

**Published:** 2023-06-29

**Authors:** Basmah M. Alharbi, Awatif M. Abdulmajeed, Alae A. Jabbour, Ahmed M. Hashim

**Affiliations:** 1 Biology Department, Faculty of Science, University of Tabuk, Tabuk 71491, Saudi Arabia; 2 Biology Department, Faculty of Science, University of Tabuk, Umluj 41912, Saudi Arabia; awabdulmajeed@ut.edu.sa; 3 Department of Biology, Faculty of Applied Sciences, Umm Al-Qura University, Makkah 21955, Saudi Arabia; aajabbour@uqu.edu.sa; 4 Department of Botany, Faculty of Science, Ain Shams University, Cairo 11865, Egypt

**Keywords:** *Avicennia marina*, antioxidant activity, Egyptian Red Sea coast, mangrove swamps, metallic pollution, oxidative damage

## Abstract

Mangrove is one of the most precious ecosystems with the greatest losses due to climate change, human activities, and pollution. The objective of this study is to assess the accumulation and distribution of some trace metals (Cu, Cd, Ni, Pb, and Zn) in sediments and *Avicennia marina* roots and leaves and to discuss the antioxidant potential of *A. marina* under metallic pollution stress. Sediments, leaf, and root samples of *A. marina* were collected from five sites along the Red Sea Coast of Egypt. Several ecological pollution indices, including the geo accumulation index (Igeo), contamination factor (CF), pollution load index (PLI), bioconcentration factor (BCF), and translocation factor (TF), were used to assess the pollution load. Cu, Cd, Ni, Pb, and Zn average concentrations in sediments were 167.4, 0.75, 110.65, 39.79, and 220 μg g^−1^, respectively, and the average values of these metals in *A. marina* roots were 44.9, 0.5, 87.96, 39.02, and 54.68 μg g^−1^, respectively, while in leaves their concentration were 50.46, 0.5572, 88.24, 40.08, and 56.08 μg g^−1^, respectively. The values of the Igeo, CF, and PLI index indicated that location 1 and 5 are moderate-to-heavily contaminated sites. On the other hand, leaves and roots of *A. marina* grown in polluted locations 1 and 5 showed high accumulation of malondialdehyde (MDA), low chlorophyll a and chlorophyll b contents concomitant with a decrease in total soluble sugars. High total antioxidant capacity was associated with a significant increase in activity levels of antioxidant enzymes (Catalase, Polyphenol oxidase, Polyphenol peroxidase, and Ascorbic acid oxidase), accumulation of secondary metabolites (total phenols, flavonoids, and tannins), and proline and carotenoids content increase. Overall, the present study suggests that the mangrove habitat of the Egyptian Red Sea coast is under the stress of anthropogenic activities, which necessitates a conservation plan to avoid further contamination and protect the unique biota of this distinctive habitat.

## 1. Introduction

Mangroves are considered an important component of intertidal wetlands due to their environmental value, which offers protection for marine fisheries, shoreline protection from erosion, and sediment stabilization. Moreover, they serve as nursery habitats for many fish species and birds, as well as protecting the coral reefs by trapping sediments derived from land [1]. Mangrove forest is one of the richest productive ecosystems on earth that provide a variety of crucial ecosystem services, especially in mitigating climate change effects [2,3,4]. Services include protection of the shoreline from floods, tsunamis, storms, and the negative effects of sea level rising through soil accretion, absorbing carbon, and storing it in the sediments (carbon sinks), sediment control, and acting as breeding grounds and shelters for marine animals including species of commercial importance. Mangroves can also provide humans with food, fuel, and construction materials [5,6,7,8].

The Red Sea encompasses two gulfs, the Suez Gulf and Aqaba Gulf, in addition to the Red Sea proper (the Red Sea main body without the two Gulfs), is shared among six countries: Yemen and Saudi Arabia on the eastern side, while Djibouti, Eritrea, Sudan, and Egypt are on the western side of it. Because the Red Sea is almost fully enclosed by land, it has a unique geography and a variety of habitats, including mangroves, coral reefs, and seagrass [9].

Along Egypt’s Red Sea coasts, mangroves cover roughly 525 hectares spread out over 28 different locations [10]. Mangroves in Egypt are primarily monospecific, comprising *A. marina* (family Avicenniaceae) (grey mangrove), except for limited locations where *Rhizophora mucronata* (family Rhizophoraceae) (red mangrove) cohabit with *A. marina* close to the Sudanese borders. Ecologically, *R. mucronata* requires humid conditions and is less tolerant and less adaptive to high salinity, little rainfall, and extreme temperatures than *A. marina* [11].

*A. marina* swamps inhabit numerous locations along the Egyptian Red Sea coast, and they are crucial to maintaining the ecological balance in different coastal habitats. Egypt’s Red Sea shoreline is vital for the provision of seafood, transportation infrastructure, and, subsequently, factors relating to public health [9]. Over the past 20 years, the mangrove forests in Egypt have faced several threats and serious perils, particularly as they experience increasing damage and decline from climate change, transportation, tourism, overfishing, deforestation, reef dredging, and marine pollution [12].

Contamination of soil, water, sediments, and even plants with trace metals is a worldwide problem [13,14]. Trace metals are released into the environment because of anthropogenic processes (industrial activities, infrastructure development, drilling and oil exploration, and shipping) or naturally because of weathering of rocks and the leaching of soils [15,16]. Trace metals that are introduced into marine habitats are stored in sediments that act as a trap for these metals and may be used to reveal trace metal pollution [17].

Mangrove swamps on the Red Sea Coast are exposed to a wide range of environmental stresses, such as high salinity, high temperatures, low oxygen levels, and metallic pollution, which in turn induce oxidative stress in mangroves via enhancing the production of reactive oxygen species [18,19]. It was reported that mangrove plants could cope with the damaging effect of ROS through a complex antioxidant system, including enzymatic antioxidants, non-enzymatic antioxidants, metal-binding proteins antioxidants, and phytonutrient antioxidants [20,21].

However, the little comprehensive literature that is available on Egyptian mangrove antioxidants’ adaptive strategies in response to trace elements pollution. Hence, the present study discusses the antioxidant potential of *A. marina* species with its specific role under metallic pollution stress. This study also aims to assess the status and distribution of trace metals in roots and leaves of *A. marina* species and their associated sediments in five locations from tidal flat zone along the Egyptian Red Sea coast.

## 2. Materials and Methods

### 2.1. Study Area Sampling

Mangrove swamps in Egypt cover about 525 hectares distributed along the coastlines of the Sinai Peninsula and the Red Sea. El-Gonah (about 25 km north of Hurghada) is the northernmost location of mangroves along the Egypt Red Sea’s mainland shore. Starting from Hurghada and heading south to the borders of Sudan (Ras Banas), mangroves are notable features in many locations, such as lagoons, bays, down streams of dry wadis, and islands [22,23].

**Figure 1:**
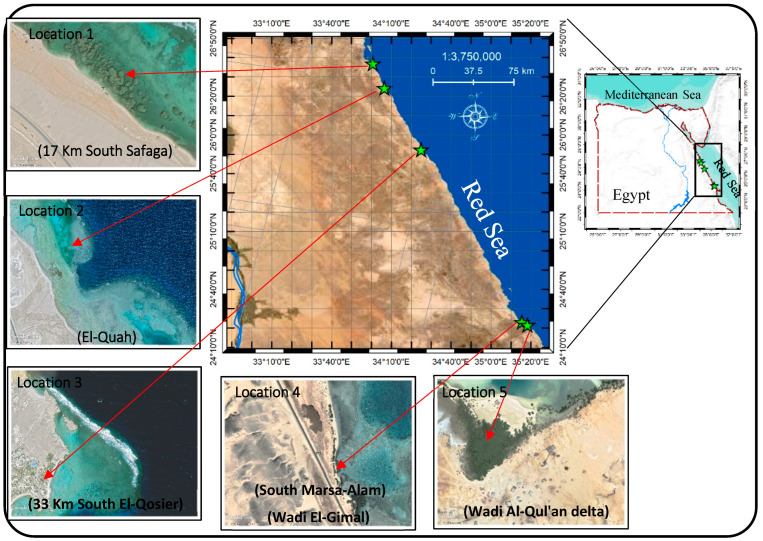
Location map for the study area along with the sampling locations and Google map images for the five selected locations.

Climatologically, the studied area (Figure 1) belongs to warm coastal deserts regions [24]. The study area’s climatic gradients from the north (in Hurghada) to the south (in Ras Banas) showed a gradual increase in temperature. The annual mean of the minimum and maximum temperatures is 17.8 °C and 27.5 °C (in Hurghada), and 19.1 °C and 32.4 °C (in Ras Banas), with a total annual mean of about 25.8 °C. Precipitation increases southward from 3 mm yr^−1^ to 17.4 mm yr^−1^. Relative humidity decreases from north (49%) to south (43%) [25,26]. *A. marina* samples (five aerial roots and five leaves) and five superficial (1–15 cm) sediment samples were collected during Spring 2022 from five locations along the study area (Figure 1). The roots and leaves samples were collected from three different individuals of *A. marina* for each location. In Table 1, specifics concerning the five locations are given, including their names, geographical coordinates, descriptions, and photos.

### 2.2. Assessment of Trace Metals in A. marina Root, Leaves, and Sediments

Sediment samples and *A. marina* root and leave samples were collected and then dried at room temperature for a week and then digested as per the standard procedures [27,28]. The sediment sample was powdered for 20 min in an electric agate mortar before being put through a 63 μm sieve and weighed after being dried overnight in an oven at 50 °C. A total of 0.2 g of each ground sample was digested for 50 min at 120–150 °C in 10 mL of a solution containing 2 mL of concentrated HNO_3_, 6 mL of HCl, and 2 mL of HF acids, and finally diluted to a total volume 50 mL with deionized H_2_O. Data are means of three replications ± SE.

Aerial roots and leave samples of *A. marina* were collected, washed with deionized water, dried for one week in the air, and then dried for 24 h at 70 °C in a hot plate. A total of 0.5 g of root and leave samples were separately digested using 20 mL of an acid mixture consisting of 750 mL of concentrated nitric acid (69%), 150 mL of concentrated sulphuric acid (98%), and 300 mL of 60% to 62% perchloric acid. Then, the solution was filtered and diluted to a volume of 50 mL by deionized H_2_O [29]. Blank sample digestion was carried out under the same conditions. Trace metals were analytically determined by inductively coupled plasma-mass spectrometry (ICP-MS) model 7500 of Agilent, USA. ICP-MS was externally calibrated. Cu, Cd, Ni, Pb, and Zn trace metal ions calibration curves were achieved using the blank and five standards (0.5, 10, 50, 100, and 200 μg/L). All the elements’ calibration curves had shown linearity, and trace metals concentrations were expressed as μg g^−1^ on the dry weight.

#### 2.2.1. Geo-Accumulation Index (Igeo)

An index of geo-accumulation (Igeo) is a quantitative indicator used to assess trace metal contamination in sediments by comparing present concentrations with the geochemical background values. According to [30], the Geo-Accumulation Index is calculated by the following equation: Igeo = Log_2_ [C_n_/(B_n_ × 1.5)], where C_n_ is the concentration of heavy metal, B_n_ is the value of geochemical background in the upper continental earth’s crust [31], and 1.5 is a constant, allowing for an analysis of the variability of trace metals because of natural processes.

#### 2.2.2. Contamination Factor (CF) and Pollution Load Index (PLI)

CF is a tool for assaying the contamination levels of a given environment. It is calculated by the following formula: CF = _Cn_/B_n_, where Cn shows measured metal contents in samples and Bn symbolizes reference or background values of metal ions without anthropogenic effects [32]. According to Hakanson [33], the following criteria were used: CF < 1 denotes little contamination; 1 to 3 indicates moderate contamination; 3 to 6 is considerable contamination; and CF > 6 is high contamination.

According to Tomlinson et al. [34], PLI is used to evaluate the degree of pollution for a certain set of metal ions at a site. The PLI values less than or equal to unity reflect the absence of pollution, whereas PLI values more than unity describe the polluted status of the site. It is calculated according to the following equation:

PLI = (CF_1_ × CF_2_ × CF_3_ ×………………….× CF_n_) 1/n.

where n is the number of trace metals analyzed in the location, and CF is the contamination factor value for each location.

#### 2.2.3. Biological Concentration Factor (BCF)

BCF is a ratio of heavy metal concentration in plant tissues to its concentration in the associated sediment sample [35]. It is calculated by the following equation:

BCF *_root_* = C _root_/C _sediment_

BCF _leaf_ = C _leaf_/C _sediment_

where C _root_, C _leaf_, and C _sediment_ are the concentration of metal ions in root, leaves, and sediment samples, respectively.

#### 2.2.4. Translocation Factor (TF)

TF is a tool used to explain the plant’s capability of absorption and distribution of trace metals through their bodies. TF is calculated by the following equation:

TF _leaf_ = C _leaf_/C _root_

where C _root_ and C _leaf_ represent the metal ion concentrations in the root and leaves, respectively [36].

### 2.3. Phytochemical and Biochemical Assay

All the organic solvents used in this investigation were Analytical Research grade (AR grade), and the chemicals utilized were all high purity that was purchased from Sigma Aldrich Chemical Co., Darmstadt, Germany. Plant samples were collected from five different locations and either kept frozen in a deep freezer (−20 °C) for extraction and estimation of photosynthetic pigments, enzymes, carbohydrates, proline, total antioxidant capacity, and malondialdehyde, or air-dried for extraction of phenolic compounds.

#### 2.3.1. Extraction and Estimation of Pigments, Total Soluble Sugars, and Malondialdehyde

The pigment content in terms of chlorophyll a, chlorophyll b, and carotenoids were extracted and calculated according to the method of Metzner and colleagues [37]. Total soluble sugars were extracted following the method of Homme et al. [38] and measured according to Loewus [39]. Following the method of Heath and Packer [40], the quantity of lipid peroxidation was assessed by counting the MDA amount generated by the thiobarbituric acid reaction.

#### 2.3.2. Extraction and Assaying Activity of Certain Enzymes

The enzymes were extracted following the method of Mukheriee and Choudhuri [41]. A total of 250 mg of fresh tissue was frozen in liquid nitrogen and then roughly crushed by a pestle in a mortar. The pulverized powder was next added to 10 mL of 100 mM phosphate buffer (KH_2_PO_4_/K_2_HPO_4_, pH 6.8), and the obtained mixture was centrifuged at 20,000× *g* for 20 min. The supernatant was diluted to a specific volume with the same buffer and utilized as an enzyme extract to measure the activity of different enzymes. Catalase activity (CAT) was assayed according to the method of Hermans and colleagues [42]. The enzyme activity was calculated as mM of H_2_O_2_/g FW/min. For the estimation of Polyphenol oxidase (PPO), a reaction mixture of 0.1 M enzyme extract, 0.1 M catechol, and 0.1 M phosphate buffer (pH 6.5) was prepared. The blank contained 0.1 mL catechol and 2.9 mL buffer. The activity of the PPO enzyme was estimated by measuring the absorbance at 420 nm [43]. Peroxidase activity was assayed according to the Kar and Mishra method [44]. Ascorbate peroxidase (APX) activity was calculated according to the method of Koricheva et al. after slight modifications [45].

#### 2.3.3. Extraction and Estimation of Total Phenols, Flavonoid, and Tannin Contents

The total phenolic molecules were extracted, and their content was determined using Malik and Singh’s technique [46]. After extraction, the total flavonoids were measured calorimetrically using the Harborne method [47] by reacting with AlCl_3_ (the concentration of total flavonoids using the quercetin’s standard curve, expressed as µg/g dry weight, was calculated). Finally, the tannins were extracted and then measured using the procedure described by Ejikeme and colleagues [48].

#### 2.3.4. Determination of Proline and Total Antioxidant Capacity (TAC)

According to the procedure described by Bates et al. [49], the amount of free proline expressed as μg/g fresh weight was determined. Total antioxidant capacity (TAC) was measured (μg/g fresh weight) following the method of Prieto and colleagues [50].

## 3. Results and Discussion

### 3.1. Trace Metals Concentrations in A. marina Sediments

In the present study, five metal ions (Cu, Cd, Ni, Pb, and Zn) were determined in the sediment samples collected from five locations along the Red Sea coast of Egypt. The analyzed trace metals concentrations are in the ranges of 45.43–330.43, 0.45–1.45, 18.28–236.23, 5.48–89.37, and 69.5–437.4 μg g^−1^ (Table 2). Results show that these trace metals are accumulated and localized in location No. 1 (17 km south of Safaga) and location No. 5 (Wadi Al-Qul’an delta) more than the other locations (2, 3, and 4), and this may be due to the proximity of location No. 1 to Safaga seaport and the various marine transport activities. As for location No. 5, it contains many tourist activities and camping sites, as well as the fine and muddy nature of its soil. For comparison purposes, the reported trace metals’ average concentration values are also given in Table 2. These readings indicated moderate to high pollution status.

The new findings from the present study are compared with the reported trace metal concentrations (average values) that have been previously discussed in other research studies (Table 3). Copper’s average concentration in the study area (167.4 μg g^−1^) is lower than the numbers recorded in the Arabian Gulf of Saudi Arabia [51]. However, it is higher than all other values reported from the Red Sea coast [52], the Gulf of Aqaba [53], the Safaga and El-Quah locations [23], and the background continental crust [54]. Other trace metals recorded in this study (Cd, Ni, Pb, and Zn) show higher average values in comparison with other numbers reported in previous studies (Table 3). Comparing results show that the study area is more contaminated than other investigated locations on the national level, and its pollution status ranges from moderately to heavily polluted.

### 3.2. Assessment of Pollution Indicators: Geo-Accumulation Index (Igeo), Contamination Factor (CF), and Pollution Load Index (PLI)

The geo-accumulation index (Igeo) is an effective tool for describing sediment quality [55]. It is used to compare the current metal concentrations with the geochemical background and identify the progressive variation of trace metals. After comparing the recorded values of Igeo, which are given in Table 4, with the (Igeo) classes as classified by Muller [56], it is noted that location 1 is moderately contaminated by Ni (1.06) and moderately to heavily polluted by Cu (2.00), Cd (2.27), Pb (2.13), and Zn (2.05). Likewise, location 5 shows moderate to heavy contamination by Pb (2.25) along with moderately polluted Cu (1.69), Cd (1.58), Ni (1.02), and Zn (1.87).

The key element to figuring out and ascertaining the contamination and pollution stages in the environmental medium is the contamination factor (CF) [32]. Moreover, the pollution load index (PLI) is also intended to be used in conjunction with the contamination factor to evaluate the level of pollution at any given location. PLI values below the unity indicate no contamination, while PLI values of one or more indicate site contamination [34]. Results in Table 5 reveal that the values of CF for Cu, Cd, Ni, Pb, and Zn range from 0.82–6, 2.25–7.25, 0.24–3.14, 0.43–7.17, and 0.99–6.24, respectively. According to CF criteria applied by Hakanson [33], location 1 is heavily contaminated by different investigated trace metals except for Ni (considerable contamination). On the contrary, location 5 is heavily contaminated by Pb and considerably contaminated by other trace metals (Table 5). While locations 2, 3, and 4 show low to moderate pollution. By evaluating the data obtained in Table 5, PLI values were found to be higher than unity (PLI > 1) in locations 1, 2, and 5, indicating moderate pollution, and less than unity in locations 3 and 4, which shows low pollution load. The higher PLI values in the present study are recorded in locations 1 (5.63) and location 5 (4.83), which is attributed to the high level of trace metal concentrations in these two sites. High values of CF accompanied by PLI values in locations 1 and 5 indicate deterioration of sediment features and accumulation of these metal ions in mangrove habitat [57]. The level of bioaccumulation of these trace metals is closely related to the mobility and availability of these metals, in addition to the chemical and physical features of the sediments, such as organic contents, sediment grain size, pH value, and their redox potential [58,59,60].

### 3.3. Trace Metals Assessment in Roots and Leaves of A. marina (BCF and TF)

Increased levels of trace metals in sediment result in higher trace metal contents in the leaves of *A. marina* at locations 1 and 5 (Table 6), which recorded 120, 1.6, 191, 93, and 107 μg g^−1^ at location 1, and 81.6, 0.94, 187, 92, and 91.5 μg g^−1^ at location 5 for Cu, Cd, Ni, Pb, and Zn, respectively. These results are in agreement with Huang and Wang [61], and it is reflected by the higher values of bioconcentration factor (BCF) of leaves than that of the root of *A. marina* (Table 7). These results are inconsistent with the findings of Alharbi and colleagues [62]. The BCF is the ratio of metal concentration in plant tissues (roots, shoots, and leaves) to the concentration in the surrounding environment [63]. The higher BCF of leaves than roots may be attributed to the high translocation factor (TF).

Generally, a value of TF greater than one indicates an acute accumulation. The TF values are higher than one at locations 1 and 5 for all the metal ions (Table 8). These observations indicate a higher metal ion accumulation tendency of leaves than roots [36,64].

The distributions of trace metals in *A. marina* leaves and roots are calculated by the bioconcentration factor (BCF), the values of more than one indicating high trace metals accumulation in the leaves and roots of *A. marina*. In the present study, *A. marina* leaves and roots are considered hyperaccumulators for Cd and Pb at locations 1 and 5 and Ni at location 2, where the BCF values for Cd, Pb, and Ni are higher than unity. It suggests an effective mechanism of detoxification or exclusion for these metals by *A. marina* [65,66], as well as that some trace metals estimated in this work, such as Cu and Zn, are essential for plant growth. Zinc and copper serve as activators and cofactors of some enzymes [67]. In addition, Cu plays an important role in photosynthesis [68]. It has been recorded that Cu concentration in the above-ground parts of *Zostera marina* and *Z. japonica* seagrasses are higher than those of underground parts [69,70]. However, high concentrations of Zn and Cu could be toxic [71]. In this context, mangroves act as a bioindicator of metal pollution due to their ability to accumulate different metal concentrations in leaves which correlates with that of sediments [66].

**Figure 2:**
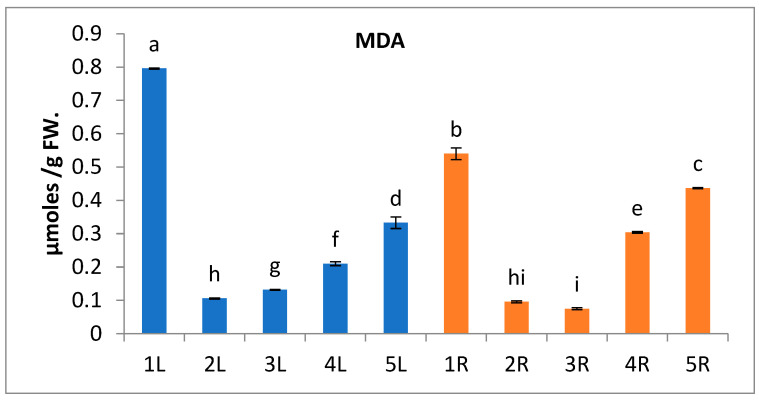
Change in malondialdehyde content of *A. marina* leaves (L) and roots (R) at different locations 1, 2, 3, 4, and 5. Each value is the mean of three replicates ± SE. Bars with different letters are significantly different at *p* ≤ 0.05.

Results in Figure 2 show that the oxidative stress caused by the trace elements contamination is correlated with the generation of ROS groups, such as superoxide radical (O_2_^−^), hydrogen peroxide (H_2_O_2_), hydroxyl radical (HO), and singlet oxygen (^1^O_2_), that resulted in lipid peroxidation of cell membrane indicated by the significant increase of malondialdehyde (MDA) levels, especially in the leaves and roots of *A. marina* inhabiting highly contaminated locations (1 and 5) [72]. Chl.a and Chl.b, on the other side, show a significant decrease at the same two locations, which may be due to the photo-oxidation and degradation processes under the effect of free radical accumulation [73].

**Figure 3:**
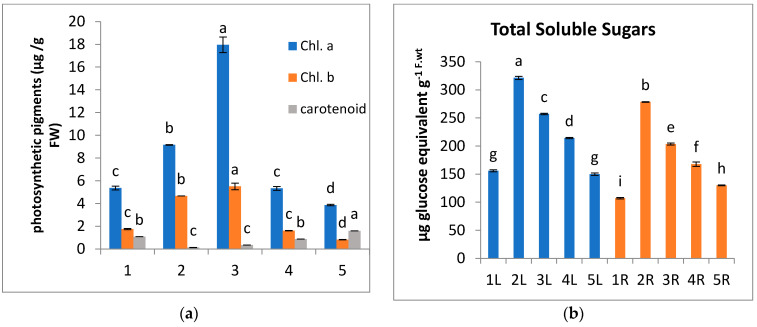
Change in (**a**) photosynthetic pigment content of *A. marina* leaves (L.) and (**b**) the total soluble sugars of *A. marina* leaves (L.) and roots (R) at different locations 1, 2, 3, 4, and 5. Each value is the mean of three replicates ± SE. Bars with different letters are significantly different at *p* ≤ 0.05.

The consequence of the decrease in photosynthetic pigments at locations 1 and 5 is that it will negatively affect the photosynthesis process, and it will be concomitant with lower sugar levels in *A. marina* leaves and roots at locations 1 and 5 (Figure 3b), which are the primary products of photosynthesis. This reduction in total soluble sugars may be due to lowered synthesis or diversion of the metabolites to other synthesis processes, such as secondary metabolites. The inhibition of photosynthesis in higher plants by trace metals has been reported by Aldoobie and Beltagi [74].

**Figure 4:**
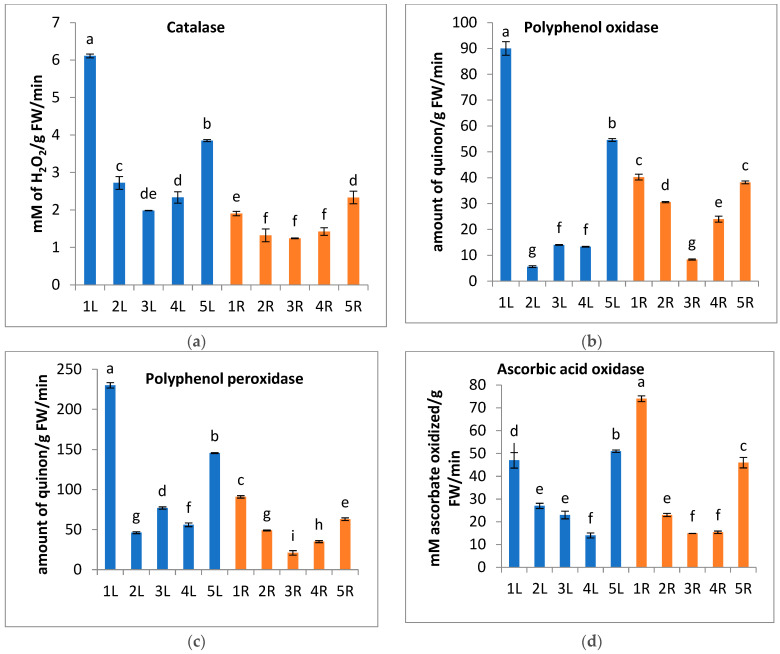
Change in the activity level of some antioxidant enzymes, (**a**) Catalase, (**b**) Polyphenol oxidase, (**c**) Polyphenol peroxidase, and (**d**) Ascorbic acid oxidase of *A. marina* leaves (L.) and roots (R) at different locations 1, 2, 3, 4, and 5. Each value is the mean of three replicates ± SE. Bars with different letters are significantly different at *p* ≤ 0.05.

In the present study, there is an increase in carotenoids contents of *A. marina* leaves at locations 1 and 5 (Figure 3a), which serve as light-harvesting pigments [75] as well as antioxidant compounds that scavenge singlet oxygen species and quench the triplet state of chlorophyll molecules [76]. The activities of four antioxidant enzymes (Catalase, Polyphenol oxidase, Polyphenol peroxidase, and Ascorbic acid oxidase) were used as biomarkers of oxidative stress in *A. marina*. The present results reveal that these antioxidant enzymes record higher activity in the leaves and roots of *A. marina* plants grown at locations 1 and 5 than in other locations. The highest values of Catalase, Polyphenoloxidase, and peroxidase are recorded in *A. marina* leaves at location 1 (Figure 4a–c), while the highest value of Ascorbic acid oxidase is (47 mM/g FW/min) in *A. marina* root at location 1 (Figure 4d). These antioxidant enzymes play an important role in scavenging ROS, such as H_2_O_2_, and removing the excess ROS to keep a relatively low and constant ROS concentration. Consequently, the self-toxicity of ROS was inhibited [77]. It was reported by Mazhoudi et al. and Mocquot et al. [78,79] that the increase in POD and CAT enzyme activity is positively related to the amounts of trace metals such as Cu, Pb, and Zn in plant tissue, and this may be attributed to the over-expression of genes encoding these enzymes [80].

**Figure 5:**
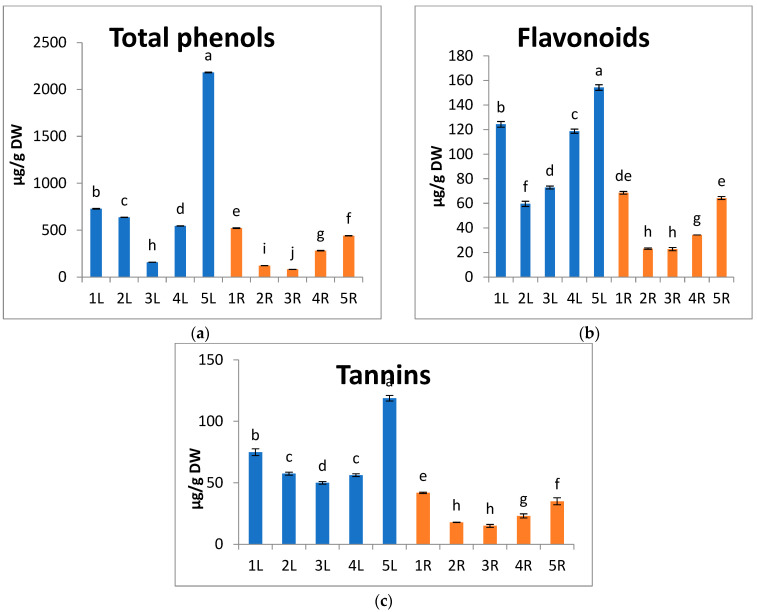
Change in (**a**) total phenols, (**b**) flavonoids, and (**c**) tannins of *A. marina* leaves (L.) and roots (R) at different locations 1, 2, 3, 4, and 5. Each value is the mean of three replicates ± SE. Bars with different letters are significantly different at *p* ≤ 0.05.

Phenolic compounds are plant secondary metabolites commonly found in plants, and they have noticed multiple biological effects, such as antioxidant activity [81]. They protect plants against stress with their high tendency to chelate metals [82]. In the current study, the total phenols, flavonoids, and tannins contents of both *A. marina* leaves and roots recorded higher values at locations 1 and 5 than at the other three locations (Figure 5a–c). The highest values recorded in leaves at location 5 are 2181.8, 154.3, and 118.8 µg/g DW for total phenols, flavonoids, and tannins, respectively. Phenolic compounds have hydroxyl and carboxyl groups and can bind with metals, particularly iron and copper [83]. The chelating ability of phenolic compounds may be related to the high nucleophilic feature of the aromatic rings rather than to certain chelating groups in the molecule [84]. Moreover, metal ions can decompose lipid hydroperoxide (LOOH) by the hemolytic break of the O-O bond and release lipid alkoxyl radicals, which induce free radical chain oxidation.

Phenolic compounds could trap this lipid alkoxyl radical and inhibit lipid peroxidation [85].

**Figure 6:**
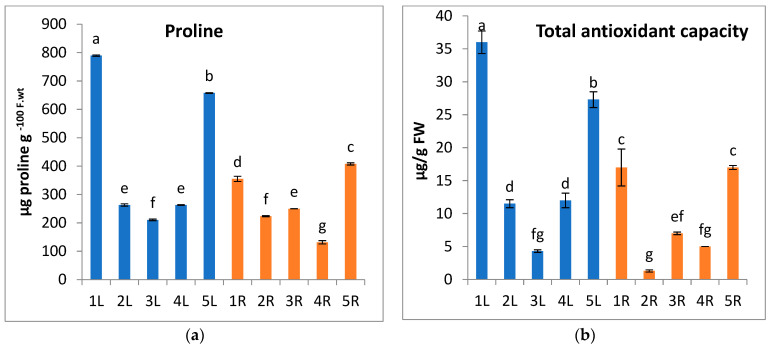
Change in (**a**) proline content and (**b**) total antioxidant capacity of *A. marina* leaves (L.) and roots (R) at different locations 1, 2, 3, 4, and 5. Each value is the mean of three replicates ± SE. Bars with different letters are significantly different at *p* ≤ 0.05.

Proline is one of the antioxidant molecules that represent the second line of defense against ROS [86]. In the present work, proline accumulation in *A. marina* shoots and roots is higher at locations 1 and 5, with the highest value (789.5 µg proline g-100 F.wt) recorded in *A. marina* leaves at location 1 (Figure 6a). It was reported by Siddique and Dubey [87] that the plants treated with toxic heavy metal content show acceleration in proline biosynthesis. Also, exogenous application of proline could suppress the heavy metal-induced lipid peroxidation as well as potassium leakage that may execute to provide protection to the plant cell [88,89].

The results show that higher enzymatic and non-enzymatic antioxidant systems are recorded in *A. marina* leaves and roots at locations 1 and 5, which is in accordance with the more pronounced levels of antioxidant capacity in leaves and roots at locations 1 and 5 with the highest value (36 µmoles/g FW) in leaves at location 1, followed by (27.3 µmoles/g FW) in leaves at location 5 (Figure 6b), indicate that *A. marina* grown at locations 1 and 5 might possess a high tolerance to metallic pollution stress.

## 4. Conclusions

In the present study, five trace metals (Cu, Cd, Ni, Pb, and Zn) were analyzed in *A. marina* roots, leaves, and their associated sediments from the tidal flat zone in five mangrove swamps along the Egyptian Red Sea Coast. The analysis indicated the following order of trace metals averages in sediments: Zn (220 μg g^−1^) > Cu (167.40 μg g^−1^) > Ni (110.65 μg g^−1^) > Pb (39.79 μg g^−1^) > Cd (0.57 μg g^−1^). These metal ions recorded the highest values in location No. 1 (17 km south of Safaga) and location No. 5 (Wadi Al- Qul’an delta), and this accumulation may be due to the industrial activities, heavy transportation in Safaga seaport (in location No. 1), human activities such as camping, and the fine muddy nature of sediments (in location No. 5). The use of different pollution indices (Igeo, CF, and PLI) to determine the pollution status indicated that location 1 is moderately polluted with Ni and moderately to heavily contaminated by Cu, Cd, Pb, and Zn. While location 5 is moderately contaminated by Cu, Cd, Ni, and Zn but heavily polluted with Pb. The values of CF for different reported trace metals heavily indicate contamination of location 1 with Cu, Cd, Pb, and Zn, but location 5 was heavily contaminated with Pb. The higher CF values in locations 1 and 5 were accompanied by high PLI values, revealing higher trace metal concentrations at these two locations. The values of BCFs of Cd and Pb in both roots and leaves of *A. marina* were more than unity in locations 1 and 5 and for Ni in location 2, representing a high accumulation of these metal ions in the roots and leaves of the mangrove. Translocation factor values were more than 1 for all reported trace metals in roots and leaves of *A. marina* in locations 1 and 5 and for Pb in location 2 only. High values of TF confirmed that *A. marina* organs could accumulate and translocate different trace metals in case of acute contamination. Results also concluded that *A. marina* roots and leaves showed higher enzymatic and non-enzymatic antioxidant activity as an adaptive response to metallic pollution on sites of heavy pollution load. Finally, the mangrove swamps located along the Egyptian Red Sea coast are exposed to many dangers and threats. We have found that some of these locations are moderately to heavily contaminated with trace metals, which in turn affect marine life and human benefits.

## Figures and Tables

**Table 1:** Specifics of the five studied locations (names, geographical coordinates, description, and field photos).

No.	Location Name	Geographical Coordinates		Description	Field Photo
		North	East		
1	17 Km South Safaga	26°36′55.9″	34°00′43.1″	It is located 17 km south of Safaga city, which has many tourist villages and the seaport of Safaga.	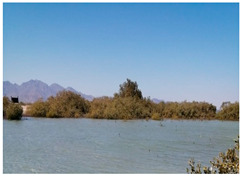
2	El-Quah	26°24′16.2″	34°06′35.2″	It is located 44 km south of Safaga City. This location has many sand dunes inhabited by *Zygophyllum* and *Tamarix* species. Mangrove swamps in this location are healthy and of high density.	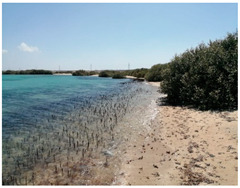
3	33 Km South El-Qosier	25°52′22.1″	34°25′02.1″	Mangrove swamps in this site are completely located inside the mangrove bay hotel (33 km south of Qosier along the road to Marsa Alam). They are protected from grazing camels.	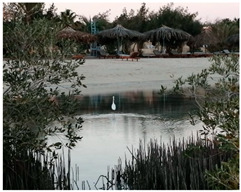
4	South Marsa-alam (Wadi El-Gimal)	24°22′58.1″	35°15′39.0″	A small stand of *A. marina* is grown in this location. Mangroves are healthy and highly protected. No grazing or human activities are noted in this location.	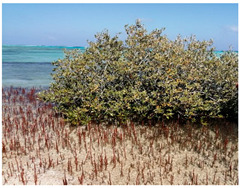
5	Wadi Al- Qul’an delta	24°21′27.1″	35°18′22.2″	This site is located in Wadi Al- Qul’an village. There are many human activities on this site, such as diving, camping, village sewage, and many solid wastes, in addition to grazing by camels.	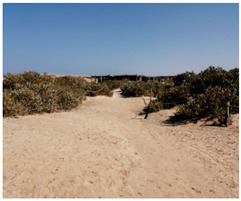

**Table 2:** Concentrations of trace metals (μg g^−1^) in *Avicennia marina* sediments collected from five studied locations along the Egyptian Red Sea Coast.

Location	Trace Metal Concentration (μg g^−1^)				
	Cu	Cd	Ni	Pb	Zn
1	330.43 ± 1.40	1.45 ± 0.03	236.23 ± 1.66	82.27 ± 0.75	437.40 ± 1.60
2	130.30± 1.28	0.45 ± 0.03	33.63 ± 1.31	15.63 ± 0.4	114.00 ± 2.00
3	63.17 ± 2.02	0.50 ± 0.02	18.28 ± 0.26	5.48 ± 0.02	69.50 ± 1.50
4	45.43 ± 1.86	0.45 ± 0.02	36.47 ± 1.50	5.92 ± 0.16	95.00 ± 2.00
5	267.50 ± 1.50	0.92 ± 0.18	229.17 ± 1.76	89.37 ± 0.95	384.30 ± 1.70
Average	167.40	0.75	110.65	39.79	220.00
Background continental crust (Taylor 1964)	55	0.20	75	12.5	70

**Table 3:** Comparison of the concentration (μg g^−1^) of trace metals (ranges and averages) in superficial sediments, as reported in the present study and other locations.

Location	Cu	Cd	Ni	Pb	Zn	Reference
Egyptian Red Sea Coast	167.40	0.75	110.65	39.79	220.00	Current study
Red Sea, Egypt	0.38	0.09	3.16	2.56	7.66	[52]
Gulf of Aqaba	7.60–10.80	0.06–0.07	----	3.7–6.8	7.00–7.70	[53]
Arabian Gulf, Saudi Arabia	182.97	0.23	75.01	5.35	52.68	[51]
17 km South Safaga	33.47	0.23	18.56	6.14	53.08	[23]
El-Quah location	35.93	0.58	30.97	5.46	68.91	[23]
Background continental crust	55	0.2	75	12.5	70	[54]

**Table 4:** Averages of geo-accumulation (Igeo) of the studied trace metals in all sites.

Sites	Averages of Igeo of the Studied Trace Metals				
	Cu	Cd	Ni	Pb	Zn
1	2.00	2.27	1.06	2.13	2.05
2	0.65	0.58	−1.74	−0.25	0.11
3	−0.37	0.73	−2.61	−1.77	−0.59
4	−0.85	0.58	−1.62	−1.68	−0.14
5	1.69	1.58	1.02	2.25	1.87

**Table 5:** Averages of contamination factor (CF) of the studied trace metals in all sites.

Sites	CF of the Studied Trace Metals					PL Index
	Cu	Cd	Ni	Pb	Zn	
1	6.00	7.25	3.14	6.58	6.24	5.63
2	2.36	2.25	0.44	1.25	1.62	1.37
3	1.15	2.50	0.24	0.43	0.99	0.79
4	0.82	2.25	0.48	0.46	1.35	0.89
5	4.86	4.50	3.05	7.17	5.49	4.83
Average	3.03	3.75	1.47	3.17	3.13	2.70

**Table 6:** Mean ± SE of trace metals concentrations (μg g^−1^) in leaves and roots of the mangrove *Avicennia marina* at different locations. Data are means of three replications ± SE, and at *p* < 0.05, cells with different letters significantly differ.

Sites	Trace Metals Concentration in Roots					Trace Metals Concentration in Leaves				
	Cu	Cd	Ni	Pb	Zn	Cu	Cd	Ni	Pb	Zn
1	90 ± 1.7 a	1.5 ± 0.2 a	180 ± 2.8 a	89 ± 1.7 a	92 ± 1.2 a	120 ± 1.7 a	1.6 ± 00.17 a	191 ± 2.9 a	93 ± 1.7 a	107 ± 3.4 a
2	27.6 ± 0.58 c	0.022 ± 0.001 c	50 ± 1.7 b	6.3 ± 0.17 b	40.5 ± 2.3 c	20.5 ± 0.6 c	0.21 ± 0.006 c	40 ± 1.1 b	7.4 ± 0.3 b	29.3 ± 2.3 c
3	19.4 ± 1.1 d	0.025 ± 0.001 c	11.3 ± 0.2 c	4.1 ± 0.06 b	31.2 ± 0.6 d	14.5 ± 0.3 c	0.015 ± 0.00 c	10.2 ± 0.12 c	4 ± 0.05 b	26.8 ± 1.3 c
4	17.5 ± 0.28 d	0.033 ± 0.002 c	15.5 ± 0.3 c	4.7 ± 0.11 b	26.7 ± 1.1 d	15.7 ± 1.2 c	0.021 ± 0.001 c	13 ± 0.6 c	4 ± 0.01 b	25.8 ± 1.7 c
5	70 ± 2.3 b	0.92 ± 0.01 b	183 ± 1.5 a	91 ± 1.7 a	83 ± 1.7 b	81.6 ± 4 b	0.94 ± 0.023 b	187 ± 1.7 a	92 ± 2.3 a	91.5 ± 2.3 b
Average	44.9	0.5	87.96	39.02	54.68	50.46	0.5572	88.24	40.08	56.08

**Table 7:** Biological concentration factors (BCF) of trace metals in mangrove *Avicennia marina* roots and leaves grown at the Egyptian African Red Sea Coast.

Sites	Bioconcentration Factors (BCF) in Roots					Bioconcentration Factors (BCF) in Leaves				
	Cu	Cd	Ni	Pb	Zn	Cu	Cd	Ni	Pb	Zn
1	0.27	1.03	0.76	1.08	0.21	0.36	1.1	0.80	1.13	0.24
2	0.21	0.04	1.49	0.40	0.35	0.28	0.06	1.19	0.47	0.25
3	0.30	0.05	0.61	0.75	0.44	0.22	0.03	0.55	0.73	0.38
4	0.38	0.07	0.42	0.80	0.28	0.51	0.04	0.35	0.68	0.27
5	0.26	1.02	0.79	1.01	0.21	0.30	1.04	0.81	1.02	0.23

**Table 8:** Translocation factors (TF) of trace metals in mangrove *Avicennia marina* roots and leaves grown at the Egyptian African Red Sea coast.

Sites	Translocation Factor (TF)				
	Cu	Cd	Ni	Pb	Zn
1	1.33	1.06	1.06	1.04	1.16
2	0.74	9.54	0.8	1.17	0.72
3	0.74	0.6	0.90	0.97	0.85
4	0.89	0.63	0.83	0.85	0.96
5	1.16	1.02	1.02	1.01	1.10

## Data Availability

The data presented in this study are available in article.
